# Factors associated with malaria microscopy diagnostic performance following a pilot quality-assurance programme in health facilities in malaria low-transmission areas of Kenya, 2014

**DOI:** 10.1186/s12936-017-2018-2

**Published:** 2017-09-13

**Authors:** Fredrick Odhiambo, Ann M. Buff, Collins Moranga, Caroline M. Moseti, Jesca Okwara Wesongah, Sara A. Lowther, Wences Arvelo, Tura Galgalo, Thomas O. Achia, Zeinab G. Roka, Waqo Boru, Lily Chepkurui, Bernhards Ogutu, Elizabeth Wanja

**Affiliations:** 1 Field Epidemiology and Laboratory Training Programme, P.O. Box 225-00202, Nairobi, Kenya; 2 Jomo Kenyatta University of Agriculture and Technology, P.O. Box 62000-00200, Nairobi, Kenya; 3 Division of Parasitic Diseases and Malaria, Center for Global Health, U.S. Centers for Disease Control and Prevention, 1600 Clifton Rd NE, Atlanta, GA 30333 USA; 4 U.S. President’s Malaria Initiative, United Nations Avenue, P.O. Box 606, Village Market, Nairobi, 00621 Kenya; 5 United States Army Medical Research Unit-Kenya, Malaria Diagnostics Center, P.O. Box 54, Kisumu, 40100 Kenya; 6 Kenya Medical Research Institute, Centre for Clinical Research, P.O. Box 1578, Kisumu, 40100 Kenya

**Keywords:** Malaria, Microscopy, Quality assurance, Interpretation, Validity, Reliability, Laboratory, Kenya

## Abstract

**Background:**

Malaria accounts for ~21% of outpatient visits annually in Kenya; prompt and accurate malaria diagnosis is critical to ensure proper treatment. In 2013, formal malaria microscopy refresher training for microscopists and a pilot quality-assurance (QA) programme for malaria diagnostics were independently implemented to improve malaria microscopy diagnosis in malaria low-transmission areas of Kenya. A study was conducted to identify factors associated with malaria microscopy performance in the same areas.

**Methods:**

From March to April 2014, a cross-sectional survey was conducted in 42 public health facilities; 21 were QA-pilot facilities. In each facility, 18 malaria thick blood slides archived during January–February 2014 were selected by simple random sampling. Each malaria slide was re-examined by two expert microscopists masked to health-facility results. Expert results were used as the reference for microscopy performance measures. Logistic regression with specific random effects modelling was performed to identify factors associated with accurate malaria microscopy diagnosis.

**Results:**

Of 756 malaria slides collected, 204 (27%) were read as positive by health-facility microscopists and 103 (14%) as positive by experts. Overall, 93% of slide results from QA-pilot facilities were concordant with expert reference compared to 77% in non-QA pilot facilities (p < 0.001). Recently trained microscopists in QA-pilot facilities performed better on microscopy performance measures with 97% sensitivity and 100% specificity compared to those in non-QA pilot facilities (69% sensitivity; 93% specificity; p < 0.01). The overall inter-reader agreement between QA-pilot facilities and experts was κ = 0.80 (95% CI 0.74–0.88) compared to κ = 0.35 (95% CI 0.24–0.46) between non-QA pilot facilities and experts (p < 0.001). In adjusted multivariable logistic regression analysis, recent microscopy refresher training (prevalence ratio [PR] = 13.8; 95% CI 4.6–41.4), ≥5 years of work experience (PR = 3.8; 95% CI 1.5–9.9), and pilot QA programme participation (PR = 4.3; 95% CI 1.0–11.0) were significantly associated with accurate malaria diagnosis.

**Conclusions:**

Microscopists who had recently completed refresher training and worked in a QA-pilot facility performed the best overall. The QA programme and formal microscopy refresher training should be systematically implemented together to improve parasitological diagnosis of malaria by microscopy in Kenya.

**Electronic supplementary material:**

The online version of this article (doi:10.1186/s12936-017-2018-2) contains supplementary material, which is available to authorized users.

## Background

In 2013, approximately 198 million cases of malaria and 584,000 deaths occurred globally, and 90% of the deaths were in Africa [1]. In 2012, Kenya had an estimated malaria mortality rate of 27.7 per 100,000 people [2]. Malaria accounted for almost 9 million outpatient visits in Kenya in 2012, which represented approximately 21% of all outpatient consultations [3].

Parasitological diagnosis is recommended by the World Health Organization (WHO) for all patients in whom malaria is suspected as part of the ‘test, treat, track’ strategy [4, 5]. Both microscopy and malaria rapid diagnostic tests (RDT) are recommended malaria diagnostic methods by the Kenya National Malaria Control Programme (NMCP) [6–8]. Although over 90% of public health facilities in Kenya had the capacity to diagnosis malaria, the proportion of facilities performing malaria microscopy, approximately 50%, has not changed in recent years [9]. Despite the high proportion of health facilities offering malaria diagnostic services, only 31% of malaria cases were confirmed by parasitological diagnosis in Kenya in 2013 [3].

Limited microscopy services in health facilities in Kenya and across sub-Saharan Africa have been attributed, in part, to limitations in the availability of equipment, supplies, working environment, training, and supervision [10–12]. Increasing and sustaining access to prompt diagnosis and effective treatment for at least 80% of the population across all levels of the health care system and epidemiological zones is a key objective of the Kenya National Malaria Strategy 2009–2017 [6]. Implementation of the national strategy included providing health facilities with microscopes and laboratory supplies and improving the skills of microscopists through formal microscopy refresher trainings at microscopy centres of excellence [6, 13].

From June to December 2013, the NMCP in coordination with the Malaria Diagnostics Center, Walter Reed Army Research Institute, initiated a pilot to operationalize the laboratory quality assurance (QA) policy and plan for malaria diagnostics in health facilities in malaria low-transmission areas [8, 14]. Malaria low-transmission areas were prioritized because of concerns surrounding over-diagnosis of malaria due to poor microscopy practices. Laboratory QA programmes have been shown to improve the diagnosis of malaria and, in particular, microscopy accuracy [13, 15]. Components of the 7-month pilot QA programme included 4 1-day visits by trained QA laboratory officers, who promoted internal QA/quality control (QC) processes, provided supportive supervision and on-job training, and cross-checked at least 10 malaria microscopy slides at each visit [8, 14, 16]. The pilot QA programme implementation is described in detail elsewhere [16]. Independently in 2013, there were other laboratory-strengthening activities ongoing in Kenya, such as malaria microscopy refresher trainings and the WHO Stepwise Laboratory Improvement Progress Towards Accreditation (SLIPTA) programme. The WHO SLIPTA framework was established to improve the quality of public health laboratories in developing countries through standardized processes to meet international accreditation [17]. In early 2014, a survey was conducted to identify factors associated with accurate malaria diagnosis by microscopy in 42 health facilities in malaria low-transmission areas of Kenya.

## Methods

### Study design and area

From March to April 2014, a cross-sectional survey was conducted in public-sector health facilities that included pilot QA programme facilities to identify factors associated with accurate malaria microscopy diagnosis in low-malaria transmission counties in Kenya. The health facilities were widely distributed in 10 (38%) of 26 low-malaria transmission counties in the Central, Eastern and Rift Valley regions and represented approximately 4% of public-sector health facilities in the 10 counties. In these counties, malaria transmission is seasonal with an estimated population-adjusted parasitaemia prevalence of <5% [18].

### Sample size and sampling procedure

A total of 42 public health facilities were selected to participate in the survey. Twenty-one facilities were part of the pilot QA programme from June to December 2013; these facilities were randomly selected from among 45 public-sector pilot QA programme facilities across 4 service-provision levels (i.e., dispensary, health centre, primary hospital, secondary hospital). The pilot QA programme was implemented in 83 health facilities (45 [54%] public-sector and 38 [46%] private-sector); facilities were selected to participate based on capacity to perform malaria microscopy and distance from the QA officers’ primary duty stations [16]. These facilities are referred to as ‘QA-pilot facilities.’ Twenty-one public health facilities of the same service-provision level and located in the same county as the QA-pilot facilities, but which did not participate in the QA-pilot programme, were also randomly selected to participate in the survey. These facilities are referred to as ‘non-QA pilot facilities.’

A total sample size of 756 malaria slides was calculated to detect a 5% difference in diagnostic accuracy between the QA-pilot and non-QA pilot facilities, assuming an index of accuracy of 90%, power of 0.80, 0.05 level of significance and finite population correction [19–21]. All facilities that consented to participate in the survey were provided with slides and requested to label and archive all slides prepared for malaria diagnosis between 1 January and 28 February, 2014. All thick-smear slides prepared for malaria diagnosis with a result recorded in the health-facility laboratory parasitology log-book and archived from 1 January to 28 February, 2014 were eligible for survey inclusion. Eighteen malaria slides were collected by the survey team from each health facility. Overall, the daily range of malaria slides prepared was 4–28 in QA-pilot and 4–52 in non-QA pilot facilities (Table 1). The number of slides collected for the survey represented <5% of all malaria slides archived from 1 January to 28 February, 2014 at each facility.

**Table 1:** Characteristics of surveyed health facilities in malaria low-transmission areas of Kenya, 2014

Characteristic	QA-pilot health facilities (N = 21)		Non-QA pilot health facilities (N = 21)	
	Number	Percentage	Number	Percentage
Health-facility level				
Primary care facilities	12	58	12	58
Dispensary	2	10	2	10
Health centre	10	48	10	48
Hospitals	9	42	9	42
Primary hospital	8	38	8	38
Secondary or referral hospital	1	4	1	4
Urban location	10	48	4	19
Participates in SLIPTA program	4	19	3	14
Microscope(s) in good optical condition	20	95	18	86
Workload >10 malaria slides per day	14	67	13	70

Characteristic	QA-pilot health facilities (N = 21)		Non-QA pilot health facilities (N = 21)	
	Median	Range	Median	Range
Number of microscopists				
Dispensary	1	–	3	2–3
Health centre	2	1–4	2	1–2
Primary hospital	7	5–12	4	2–7
Secondary or referral hospital	7	–	7	–
Malaria slide workload per day				
Dispensary	22	5–28	30	7–52
Health centre	17	5–21	15	4–19
Primary hospital	21	7–28	18	6–52
Secondary or referral hospital	9	4–16	18	7–28

*QA* quality assurance, *SLIPTA* stepwise laboratory improvement towards accreditation, an external laboratory-strengthening program sponsored by World Health Organization

From the slide boxes, 9 positive and 9 negative slides were collected per facility via simple random sampling using a random number table where the sequence boundary was the number of slides archived at each facility. Slides which were found unlabelled (i.e., no date, laboratory number, patient age, or sex), stuck together, not entered in the log-book or with results that were not signed by the examining microscopist were excluded. At facilities with fewer than 9 positive slides, all the positive slides were selected and the balance was randomly selected from negative slides to total 18 per facility.

### Data collection

Each microscopist who had examined the selected slides was interviewed by trained survey staff using a standardized, pilot-tested structured questionnaire (Additional file 1). Laboratory and facility conditions were collected via a standardized form (Additional file 2). Recent training for microscopists was defined as having attended initial or refresher malaria microscopy training within the year prior to the survey.

Thick-blood smear slides were examined for the presence or absence of parasites by expert microscopists who had been certified through the WHO External Competency Assessment for Malaria Microscopy scheme. Two independent expert microscopists cross-checked each of the slides and a third independent expert microscopist was a tie-breaker when the first two expert readers disagreed. Expert readers disagreed on 9 (1%) slides requiring a third tie-breaker. The expert microscopist results, or the tie-breaker result when necessary, were considered the reference value. Expert microscopists were masked to both the health-facility microscopy results and the other expert microscopy results. Expert microscopists examined a minimum of 100 high-power magnification fields before the slide was classified as negative per national and WHO guidance [8, 10]. Each microscopist read a maximum of 20 slides per day. Accurate malaria diagnosis was defined as concordance in the presence or absence of parasites (i.e., positive or negative) between the health-facility microscopist result and the expert reference result. The health-facility results were compared to expert reference to obtain validity and reliability performance measures.

### Data management and analysis

Data were entered into Excel 2010 (Microsoft, Seattle, WA, USA). The sensitivity, specificity, positive predictive value (PPV) and negative predictive value (NPV) of the health-facility microscopy results were calculated with 95% confidence intervals (CI) using exact method by Graph Pad Prism version 5.01 (GraphPad Software, La Jolla, CA, USA). Inter-reader agreement for facilities *versus* reference values was expressed as kappa (κ) values with 95% CIs using Graph Pad Prism version 5.01 [22]. Using accurate malaria diagnosis as the outcome of interest, multivariable logistic regression with institutional-specific random effects was performed using Stata version 12 (StataCorp LP, College Station, TX, USA). Both individual (i.e., recent microscopy refresher training status, level of initial training, years and location of work experience, and malaria knowledge) and institutional-level factors (i.e., participation in pilot QA programme or other external QA programme, condition of microscopes, number of microscopists, service-provision level, location and daily workload) were included as independent variables in the regression model.

### Ethical review

The study was approved by the ethical review committee of the Jaramogi Oginga Odinga Teaching and Referral Hospital (#01713, ref: ERC 1B/VOL.1/70) in collaboration with the Ministry of Health. The study underwent human subject review at CDC and was approved as non-engagement in human subject research. The management official at each health facility and each microscopist provided written consent. No personal identifiers were collected from microscopists or extracted from laboratory or clinical records.

## Results

All selected heath facilities agreed to participate in the survey. Participating health facilities were located in 10 (38%) of 26 low-malaria transmission counties. Among surveyed facilities, 58% were primary care facilities (i.e., dispensaries [10%] and health centres [48%]) and 42% were hospitals (i.e., primary [38%] and secondary or referral [4%]) (Table 1). More QA-pilot facilities were in urban settings (48 vs 19%), participated in an external laboratory-strengthening program (i.e., SLIPTA) (19 vs 14%), and had microscopes in good optical condition (95 vs 86%) compared to non-QA pilot facilities. The number of microscopists per facility and daily malaria slide workloads were similar across surveyed facilities (Table 1). As shown in Table 2, more microscopists in QA-pilot facilities had completed recent refresher training (68 vs 29%), had worked in a malaria high-transmission area (63 vs 21%), and had knowledge of national malaria diagnostic and treatment guidelines (84 vs 39%).

**Table 2:** Characteristics of surveyed microscopists in malaria low-transmission areas of Kenya, 2014

Characteristic	Microscopists at QA-pilot health facilities (N = 56)		Microscopists at non-QA pilot health facilities (N = 82)	
	Number	Percentage	Number	Percentage
Individual level				
Training and work experience				
Recent microscopy refresher training	38	68	24	29
More than diploma-level initial training	45	80	59	72
≥5 years of work experience	49	88	66	80
Worked in malaria high-transmission area	35	63	17	21
Knowledge				
Malaria diagnostic and treatment guidelines	47	84	32	39
Malaria epidemiology in county	55	98	70	85
Malaria case importation	55	98	75	91

*QA* quality assurance; recent training was defined as in the year prior to the survey

**Fig. 1:**
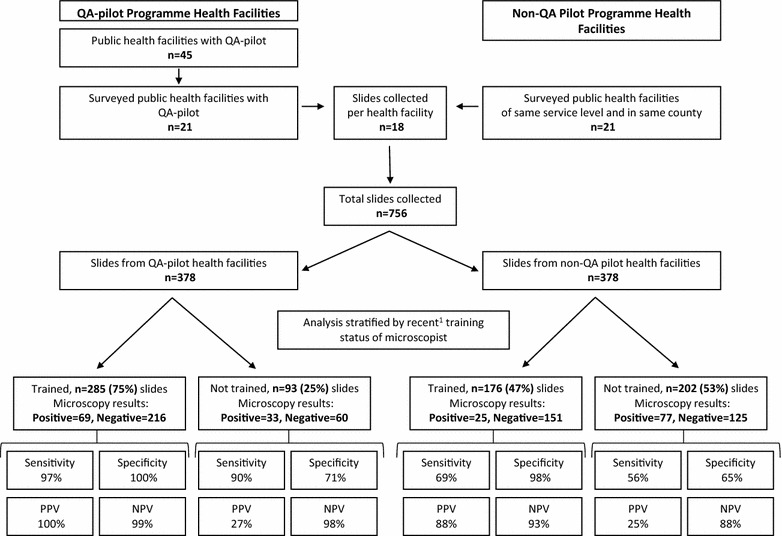
Malaria microscopy performance stratified by pilot QA programme participation and recent training status of microscopists in malaria low-transmission areas of Kenya, 2014

A total of 756 malaria slides were collected from the health facilities surveyed; 204 (27%) slides were read as positive for malaria by health-facility microscopists and 103 (14%) as positive by expert microscopists. In Fig. 1, slides are stratified by facility QA-pilot programme participation and recent training status (i.e., formal initial or refresher microscopy training within the year prior to the survey) of the microscopists. More microscopists (68%, 38 of 56) had completed recent refresher training in the QA-pilot facilities compared to non-QA pilot facilities (29%, 24 of 82) (p < 0.01). In QA-pilot facilities, recently-trained microscopists read 285 (75%) slides compared to 176 (47%) in the non-QA pilot facilities (p < 0.001). Recently-trained microscopists in QA-pilot facilities performed better on all microscopy performance measures with 97% sensitivity and 100% specificity compared to recently-trained microscopists in the non-QA pilot facilities with 69% sensitivity and 98% specificity (p < 0.01). Microscopists without recent microscopy refresher training performed the same regardless of facility participation in the QA-pilot programme.

Overall as shown in Table 3, QA-pilot facilities performed significantly better on measures of diagnostic accuracy (i.e., sensitivity, specificity, PPV and NPV) against expert reference compared to non-QA pilot facilities. The overall inter-reader agreement between QA-pilot facilities and expert microscopy was κ = 0.80 (95% CI 0.74–0.88) compared to κ = 0.35 (95% CI 0.24–0.46) in non-QA pilot facilities (p < 0.001). Table 3 also shows the diagnostic performance measures stratified by service level; only primary hospitals participating in the pilot QA programme performed statistically better on all diagnostic accuracy measures compared to non-QA pilot facilities. In total, 351 (93%) slide results were read as concordant with expert reference from QA-pilot facilities compared to 292 (77%) in the non-QA pilot facilities (p < 0.001) (Table 3).

**Table 3:** Measures of malaria microscopy performance in surveyed health facilities in malaria low-transmission areas of Kenya, 2014

Quality-assurance pilot programme	Number of slides	Sensitivity		Specificity		Positive predictive value		Negative predictive value		Kappa value	
		%	95% CI	%	95% CI	%	95% CI	%	95% CI	ĸ	95% CI
Overall											
Yes	378	*96*	(*90*–*99*)	*92*	(*88*–*95*)	*76*	(*67*–*84*)	*99*	(*97*–*99*)	*0.80*	(*0.74*–*0.88*)
No	378	62	(49–74)	80	(76–85)	40	(31–50)	91	(87–94)	0.35	(0.24–0.46)
Dispensary											
Yes	36	100	(59–100)	*100*	(*88*–*100*)	*100*	(*59*–*100*)	100	(88–100)	1.00	–
No	36	62	(24–91)	54	(34–72)	28	(10–53)	83	(59–96)	0.11	(−0.16 to 0.38)
Health centre											
Yes	180	88	(69–97)	90	(85–94)	60	(42–75)	98	(94–100)	0.65	(0.51–0.79)
No	180	59	(39–76)	94	(89–97)	65	(44–84)	92	(87–96)	0.55	(0.38–0.72)
Primary hospital											
Yes	144	*100*	(*92*–*100*)	*93*	(*85*–*97*)	*87*	(*75*–*95*)	*100*	(*96*–*100*)	*0.89*	(*0.82*–*0.97*)
No	144	62	(41–80)	69	(59–77)	30	(18–44)	89	(81–95)	0.22	(0.06–0.37)
Secondary hospital											
Yes	18	100	(40–100)	93	(66–100)	80	(28–99)	100	(75–100)	0.85	(0.58–1.00)
No	18	100	(29–100)	87	(60–98)	60	(15–95)	100	(75–100)	0.68	(0.29–1.00)

Italic denotes statistical significance

*CI* Confidence interval

In unadjusted logistic regression analysis shown in Table 4, all the microscopist characteristics were associated with accurate malaria diagnosis except initial level of training, but only pilot QA programme participation and good optical condition of microscopes were institutional factors associated with accurate malaria diagnosis. In adjusted multivariable logistic regression analysis, recent microscopy refresher training (prevalence ratio [PR] = 13.8; 95% CI 4.6–41.4), ≥5 years of work experience (PR = 3.8; 95% CI 1.5–9.9), and pilot QA programme participation (PR = 4.3; 95% CI 1.0–11.0) were the only factors significantly associated with accurate malaria diagnosis.

**Table 4:** Individual and institutional characteristics associated with accurate malaria microscopy diagnosis in surveyed health facilities in malaria low-transmission areas of Kenya, 2014

Characteristic		Slides n (%)	Accurate diagnosis n (%)	Unadjusted		Adjusted	
				Prevalence ratio	95% confidence interval	Prevalence ratio	95% confidence interval
Individual							
Recent microscopy refresher training	No	295 (39.0)	197 (66.8)	1.00 (Ref)		1.00 (Ref)	
	Yes	461 (61.0)	446 (96.7)	*40.5*	*15.1*–*108.6*	*13.8*	*4.6*–*41.4*
More than diploma-level initial training	No	141 (18.7)	109 (77.3)	1.00 (Ref)			
	Yes	615 (81.3)	534 (86.8)	2.1	0.9–4.6		
≥5 years of work experience	No	137 (18.1)	78 (56.9)	1.00 (Ref)		1.00 (Ref)	
	Yes	619 (81.9)	565 (91.3)	*23.7*	*9.7*–*57.6*	*3.8*	*1.5*–*9.9*
Worked in malaria high-transmission area	No	383 (50.7)	290 (75.7)	1.00 (Ref)			
	Yes	373 (49.3)	353 (94.6)	12.1	5.2–28.3		
Knowledge of malaria diagnostic and treatment guidelines	No	194 (25.7)	119 (61.3)	1.00 (Ref)			
	Yes	562 (74.3)	524 (93.2)	25.6	10.4–62.9		
Knowledge of malaria epidemiology in county	No	94 (12.4)	45 (47.9)	1.00 (Ref)			
	Yes	662 (87.6)	598 (90.3)	22.8	8.7–60.3		
Knowledge of malaria cases importation	No	63 (8.3)	20 (31.7)	1.00 (Ref)			
	Yes	693 (91.7)	623 (89.9)	21.1	7.2–62.1		
Institutional							
Quality-assurance pilot programme	No	378 (50.0)	292 (77.2)	1.00 (Ref)		1.00 (Ref)	
	Yes	378 (50.0)	351 (92.8)	*6.0*	*1.9*–*18.9*	*4.3*	*1.0*–*11.0*
Good optical condition of microscope(s)	No	72 (9.5)	46 (63.9)	1.00 (Ref)			
	Yes	684 (90.5)	597 (87.3)	7.6	1.1–51.4		
Rural location	No	505 (66.8)	415 (82.2)	1.00 (Ref)			
	Yes	251 (33.2)	228 (90.8)	2.7	0.7–10.1		
Participation in SLIPTA program	No	647 (85.6)	539 (83.3)	1.00 (Ref)			
	Yes	109 (14.4)	104 (95.4)	4.8	0.8–28.8		
>3 laboratory staff	No	361 (47.8)	308 (85.3)	1.00 (Ref)			
	Yes	395 (52.2)	335 (84.8)	1.1	0.3–3.3		
Hospital-level facility	No	432 (57.1)	376 (87.0)	1.00 (Ref)			
	Yes	324 (42.9)	267 (82.4)	0.8	0.2–2.5		
Workload >10 slides per day	No	269 (35.6)	239 (88.8)	1.00 (Ref)			
	Yes	487 (64.4)	404 (83.0)	0.6	0.2–2.1		

Italic denotes statistical significance; recent training was defined as in the year prior to the survey

*SLIPTA* stepwise laboratory improvement towards accreditation, sponsored by World Health Organization; *Ref* reference

## Discussion

This observational study demonstrated that diagnostic accuracy of malaria microscopy was positively associated with recent microscopy refresher training and ≥5 years of experience for microscopists and health facility participation in the pilot QA programme. The findings are consistent with other studies from Kenya and elsewhere that have shown both laboratory QA programmes and microscopy refresher trainings improve malaria microscopy performance [13, 15, 23–25]. In 2013, the NMCP independently started both formal refresher trainings for microscopists at a malaria microscopy centre of excellence and the pilot QA programme for malaria diagnostics at 83 health facilities; both the refresher trainings and pilot QA programme were intended to improve malaria diagnosis by microscopy. However, implementation of the two diagnostic strengthening components was not coordinated or systematic in health facilities, across service-provision levels or administrative zones, which hindered independent evaluation of the pilot QA programme.

Recent microscopy refresher training at the individual level was more strongly associated with accurate malaria diagnosis than health facility participation in the pilot QA programme. However, microscopists who had recently completed refresher training and worked in a facility that was part of the pilot QA programme had the best performance for all measures of diagnostic accuracy. These findings suggest that synergies exist between formal microscopy refresher training and the pilot QA programme. Implementation of both diagnostic strengthening components together appear to produce the best performance results. Therefore, the NMCP and partners should consider systematically implementing formal microscopy refresher training and the QA programme together as a package of interventions to improve parasitological diagnosis of malaria by microscopy in accordance with national and WHO guidance [8, 10, 14, 26].

Malaria microscopy refresher training was an important confounder in the study. The study was powered to detect differences at the health-facility level rather than at the individual microscopist level, and malaria microscopy refresher training was not uniform across surveyed facilities. Twice as many microscopists from QA-pilot facilities had recent refresher training compared to non-QA pilot facilities. Three-quarters of the malaria slides from QA-pilot facilities were read by microscopists who had recently completed malaria microscopy refresher training compared to less than half of the slides from non-QA pilot facilities. In addition, there were other general laboratory-strengthening activities ongoing, such as SLIPTA, in a minority of facilities that were included in the survey. Although participation in the WHO SLIPTA programme was not significantly associated with accurate malaria microscopy diagnosis, the programme might have contributed to overall laboratory improvements that were not specifically measured [17].

Overall in QA-pilot facilities, the sensitivity, specificity and NPV were very high at over 90%. The PPV was much lower, but lower PPVs and higher NPVs would be expected because all the surveyed facilities were located in malaria low-transmission counties. These counties have community malaria parasitaemia prevalences by microscopy of between 1 and 3% during peak malaria transmission season [27]. A 2014 national health-facility survey for malaria infection found that 3.4% of outpatients who reported a history of fever within the last 48 h had a positive malaria RDT in seasonal low-transmission counties in Kenya [28]. Malaria slides were collected in January and February for the survey, which is not the peak malaria transmission season in Kenya. Therefore, most persons presenting to health facilities, even if febrile, were unlikely to have malaria at the time of the survey. In malaria low-transmission settings, the low PPV findings translate into a large number of false-positive results. Persons misdiagnosed as having malaria when they do not are at risk of not being treated for their actual illness, which can lead to increased morbidity and potentially mortality. In addition, treating people who do not have malaria with relatively expensive artemisinin-based combination therapy wastes limited resources and can contribute to the development of artemisinin resistance [4, 7, 10, 26].

Hospitals require expert microscopy for the management of complicated patients with severe malaria and co-morbidities. Expert microscopy is the gold standard for identifying mixed infections, treatment failures, and quantifying parasite density [8, 10, 26]. Hospitals generally have more substantial laboratories and resources available to maintain at least adequate, if not expert, diagnostic microscopy programmes compared to outpatient health centres and dispensaries. Outpatient health centres and dispensaries generally have high patient workloads, which makes labour-intensive diagnostics, such as malaria microscopy, challenging. Historically in Kenya, programmes and training cascaded from the highest service-provision levels to the lowest and often did not reach dispensaries due to limited resources and lower prioritization. In 2010, Kenya prioritized dispensaries to receive malaria RDTs for parasitological diagnosis since expert microscopy services were not expected at this level [3].

However, the strategy for utilizing malaria RDTs and microscopy concurrently to improve diagnostic performance across service levels and malaria epidemiologic zones is not clear in the national diagnostic and treatment guidelines [6–8].

This study has a number of limitations. Although health facilities were randomly selected for the survey, the facilities selected to participate in the pilot QA programme were a convenience sample. Thus, the surveyed facilities are not representative of all public health facilities in Kenya, which limits the generalizability of the findings. A baseline evaluation of microscopy performance was not conducted prior to the start of the refresher trainings or the pilot QA programme. Microscopists and facilities selected for participation in the diagnostic strengthening components might have performed better at baseline compared to those not selected. Therefore, the association between microscopy performance and refresher training and the pilot QA programme might have been overestimated. Additionally, when health facilities consented to participate in the survey, they were asked to store slides during a specific time interval for later retrieval. Facilities might have preferentially stored slides for which they felt confident about the results, and microscopists might have performed better during this period because they were aware of the survey (i.e., Hawthorne effect) [29, 30]. Both situations would have resulted in an overestimation of diagnostic accuracy, but the potential bias should be non-differential across all facilities.

Another important limitation was that slide preparation quality, including the stain type and adequacy, was not evaluated. Although both NMCP and WHO recommend Giemsa preferentially for malaria microscopy, the use of both Giemsa and Field stains was common in health facilities [8, 10, 16]. Slides were not matched on parasite density either. Thick films were examined for the presence or absence of parasites; no thin films were examined for parasite density or speciation [8, 10]. Slides from QA-pilot facilities might have had higher parasite densities, which would make malaria easier to identify correctly. However, urban areas generally have a substantially lower parasitaemia prevalence compared to rural areas and a greater percentage of QA-pilot facilities were located in urban areas [21, 22]. Therefore, it is possible that persons who presented to QA-pilot facilities in urban areas would have had lower parasite densities overall; if this represented the true situation, then QA-pilot enrolled facilities would have performed better than estimated compared to non-QA pilot facilities.

## Conclusions

Diagnostic accuracy of malaria microscopy was positively associated with recent microscopy refresher training and ≥5 years of experience for microscopists at the individual level and pilot QA programme participation at the health-facility level. Microscopists who had recently completed refresher training and worked in a QA-pilot facility had the best performance for all measures of diagnostic accuracy. Therefore, formal microscopy refresher training and the QA programme should be systematically implemented together to improve parasitological diagnosis of malaria by microscopy in Kenya.

## Additional files

**Additional file 1.** Microscopist questionnaire.

**Additional file 2.** Health facility information and slide collection form.
