# Supplementary material for: Factors associated with malaria microscopy diagnostic performance following a pilot quality-assurance programme in health facilities in malaria low-transmission areas of Kenya, 2014
Source: Malar J. 2017 Sep 13;16:371. doi: 10.1186/s12936-017-2018-2 (PMC5598012; doi:10.1186/s12936-017-2018-2)
Supplement: Supplementary file 1 — Additional file 1. Microscopist questionnaire. [file 12936_2017_2018_MOESM1_ESM.docx]

**QUESTIONNAIRE FOR MICROSCOPIST LEVEL INFORMATION**

**Health facility identification number (***use the assigned unique code***) -------------------------------**

**Microscopist ID (***use the assigned unique code in the slide collection form****)* ------------------------**

***Age (****yy****)*** ---------------------------------------------------------------------------------------------------------

***Sex (****M/F****)* -------------------------------------------------------------------------------------------------------**

**Date of interview----------------------------------------------------------------------------------------------**

**Interviewer’s name ------------------------------------------------------------------------------------------**

**Education level**

1. Are you professionally trained in medical laboratory science (*Yes/No*)? --------------------------
2. If *YES,* what was your initial training (Tick all that applies to you)?
3. Certificate--------------------------Date of completion (*mm/yy*)-----------------------------------
4. Diploma-----------------------------Date of completion (*mm/yy*) ---------------------------------
5. Higher Diploma-------------------Date of completion (*mm/yy*) ----------------------------------
6. Degree ------------------------------Date of completion (*mm/yy*) ---------------------------------
7. Others (Specify)--- ---------------Date of completion (*mm/yy*) -------------------------------

**Work experience**

1. When were you first employed to practice as a medical laboratory scientist (*mm/yy*)? -------
2. In your opinion, is it possible that *SOME* of the malaria cases reported in this health facility are from infections acquired from other malaria epidemiological areas (*Yes/No*)? ------------
3. If Yes, and giving reasons, which of the 47 counties would the infection have been imported from (*at most counties*)?
4. County Reason(tick if applicable) Other reasons (s*pecify*)

High-transmission, endemic

High-transmission, endemic

High-transmission, endemic

High-transmission, endemic

High-transmission, endemic

1. In your duty, how many years have you been using microscopy in the diagnosis of malaria (yy)? --------------------------------------------------------------------------------------------------------
2. Have you used methods other than microscopy in the diagnosis of malaria in your duty (Yes/No)? -------------------------------------------------------------------------------------------------
3. If Yes, which of the following methods (*tick all that apply to you*):
4. Rapid diagnostic tests (RDTs) to detect specific parasite antigens
5. Detection of parasite DNA by polymerase chain reaction (PCR)
6. Rapid diagnostic tests (RDTs) to detect specific parasite antibodies
7. Others (*specify*) ------------------------------------------------------------
8. State the approximate duration you have used the above declared methods (mm/yyyy-mm/yyyy).
9. Have you ever worked in malaria high-transmission area(s) (*Yes/No*)? -----------------------
10. If *Yes* provide the following

| Health Facility name | County/District | Period (*mm/yy-mm/yy*) | Comment |
| --- | --- | --- | --- |
|  |  |  |  |
|  |  |  |  |
|  |  |  |  |
|  |  |  |  |
|  |  |  |  |

**Refresher training**

1. Have you attended any malaria microscopy refresher training between 01January-31 December 2013(*Yes/No*) -----------------------------------------------------------------------------
2. If *YES* provide the following:

A.

1. Name or certification of the course attended -------------------------------------------------
2. Date of the training (mm/yy)-------------------------------------------------------------------
3. Duration in hours of the training ---------------------------------------------------------------
4. Provider of the training --------------------------------------------------------------------------
5. Venue/centre of the training --------------------------------------------------------------------

B.

1. Name or certification of the course attended -------------------------------------------------
2. Date of the training (mm/yy)-------------------------------------------------------------------
3. Duration in hours of the training ---------------------------------------------------------------
4. Provider of the training --------------------------------------------------------------------------
5. Venue/centre of the training --------------------------------------------------------------------

C.

1. Name or certification of the course attended -------------------------------------------------
2. Date of the training (mm/yy)-------------------------------------------------------------------
3. Duration in hours of the training ---------------------------------------------------------------
4. Provider of the training --------------------------------------------------------------------------
5. Venue/centre of the training --------------------------------------------------------------------
6. If *No*, have you ever attended a malaria microscopy refresher training in your life (*Yes/No*) -----------------------------------------------------------------------------------------

If *YES* provide the following:

A.

1. Name or certification of the course attended -------------------------------------------------
2. Date of the training (mm/yy)-------------------------------------------------------------------
3. Duration in hours of the training ---------------------------------------------------------------
4. Provider of the training --------------------------------------------------------------------------
5. Venue/centre of the training --------------------------------------------------------------------

B.

1. Name or certification of the course attended -------------------------------------------------
2. Date of the training (mm/yy)-------------------------------------------------------------------
3. Duration in hours of the training ---------------------------------------------------------------
4. Provider of the training --------------------------------------------------------------------------
5. Venue/centre of the training --------------------------------------------------------------------

C.

1. Name or certification of the course attended -------------------------------------------------
2. Date of the training (mm/yy)-------------------------------------------------------------------
3. Duration in hours of the training ---------------------------------------------------------------
4. Provider of the training --------------------------------------------------------------------------
5. Venue/centre of the training --------------------------------------------------------------------

**Guideline Knowledge**

1. Have you seen this current ‘NATIONAL GUIDELINES FOR THE DAIGNOSIS, TREATMENT AND PREVENTION OF MALARIA IN KENYA, Fourth Edition”(*show a copy*) (*Yes/No*) ---------------------------------------------------------------------------------------
2. Have you read this current ‘NATIONAL GUIDELINES FOR THE DAIGNOSIS, TREATMENT AND PREVENTION OF MALARIA IN KENYA, Fourth Edition” (*Yes/No*)-----------------------------------------------------------------------------------------------
3. The current ‘NATIONAL GUIDELINES FOR THE DAIGNOSIS, TREATMENT AND PREVENTION OF MALARIA IN KENYA, Fourth Edition” recommends the following regarding parasitological diagnosis and treatment of uncomplicated malaria (*Indicate FALSE or TRUE in the boxes against each statement* ):
4. Patients presenting with signs and symptoms of uncomplicated malaria should be tested for malaria
5. Only those who test positive should be treated for malaria
6. Patients should also be assessed for other conditions that may cause fever and be managed accordingly
7. Children aged below 5 years presenting with signs and symptoms should be treated regardless of the test results
8. Appropriate treatment should never be delayed or denied due to inability to test for malaria
9. In your understanding, what are the commonly used routine ‘parasitological diagnostic’ methods of malaria in Kenya?
10. Microscopy, Rapid diagnostic tests (RDTs) to detect specific parasite antigens
11. Microscopy, Rapid diagnostic tests (RDTs) to detect specific parasite antibodies
12. Detection of parasite DNA by polymerase chain reaction (PCR), Rapid diagnostic tests (RDTs) to detect specific parasite antigens
13. Detection of parasite DNA by polymerase chain reaction (PCR), Rapid diagnostic tests (RDTs) to detect specific parasite antibodies
14. Rapid diagnostic tests (RDTs) to detect specific parasite antigens, Rapid diagnostic tests (RDTs) to detect specific parasite antibodies

**Epidemiology knowledge**

1. Kenya has how many malaria epidemiological zones (*tick one*)?
2. Three
3. Four
4. Five
5. Six
6. Seven
7. In which of the following epidemiological zones does this county belong (*tick one*)?
8. Endemic
9. Seasonal
10. Epidemic prone areas of western highlands of Kenya
11. Low risk malaria areas
12. What is the community prevalence of *Plasmodium falciparum* in this county (*tick one*)?
13. <0.1%
14. 0.1%-<1.0%
15. 1.0%-<5.0%
16. 5.0%-<10.0%
17. 10.0%-<20.0%
18. 20.0%-<40.0%
19. >=40.0%
