# Supplementary material for: Factors associated with malaria microscopy diagnostic performance following a pilot quality-assurance programme in health facilities in malaria low-transmission areas of Kenya, 2014
Source: Malar J. 2017 Sep 13;16:371. doi: 10.1186/s12936-017-2018-2 (PMC5598012; doi:10.1186/s12936-017-2018-2)
Supplement: Supplementary file 2 — Additional file 2. Health facility information and slide collection form. [file 12936_2017_2018_MOESM2_ESM.pdf]

STANDARDIZED FORM FOR FACILITY INFORMATION AND SLIDES COLLECTION

1. HEALTH FACILITY INFORMATION

Health facility identification number (*use the assigned unique code***)** --

Health facility service level (*observe the service charter and confirm with management: 2, 3, 4, 5****)*** ----------------------------------

Address: P.O.Box --

E-mail---

QA-pilot implementation status (*Yes/No****)*** ---

Region (*Central, Eastern, Rift Valley****)*** ---

County--

Epidemiological zone---***Location (****Urban/Rural****)*** --

Head of health facility (*name facility in-charge or medical superintendent***)** ---

----

E-mail --**Tel** --

Head of Laboratory --

E-mail --**Tel** --

SLAMTA/SLIPTA/ WHO-AFRO Registration (*Yes/No*) ------ Other accreditation body (*specify*) ---------------------------------

Number of practicing Lab. techs in the health facility laboratory --

Date of visit/ data collection ---

Data collected by --

2. DOCUMENTS AND RECORDS

Confirm availability of the following:

**Quality essentials**	**Y**	**P**	**N**
1. Malaria blood film (MBF) sample collection SOP present.			
2. MBF preparation SOP present.			
3. MBF reading and quantification SOP present.			
4. MBF staining SOP present.			
5. Species reference charts (micrographs).			
6. Staining procedure job aid.			
7. Parasite quantification job aid.			
8. Giemsa stain preparation SOP present.			
9. PH 7.0-7.2 buffer preparation SOP present.			
10. Internal quality control for stains documented.			
11. Microscope maintenance logs complete.			
12. Test results logbook complete.			
13. Occurrence book available and complete.			
14. Slide easily traceable			

3. SLIDE INFORMATION

1. Microscope brand in-use--

i. Optical condition of microscope in-use (*team to examine high-power magnification fields of stained malaria slide sample to confirm: Good=1, Bad=2*) --

2. Turnaround time (*as stated in the Public service charter*) ---

3. Average workload (*total for Jan & Feb 2014 divided by total number of working days*) ---------------------------------/day

Slide collection form:

**Slide no.**	**Date exam**	**Health facility microscopist's result (positive, Negative)**	**Assigned code for microscopist/slide examiner (*use the assigned unique code*)**
